# Study on mechanical properties of interbedded rock masses with microcrack based on thermal-mechanical coupling

**DOI:** 10.1371/journal.pone.0280486

**Published:** 2024-02-23

**Authors:** Liewang Qiu, Liangfu Xie, Yongjun Qin, Jianhu Wang, Shan Liu, Jiangu Qian

**Affiliations:** 1 College of Civil Engineering and Architecture, Xinjiang University, Urumqi, China; 2 Xinjiang Civil Engineering Technology Research Center, Urumqi, China; 3 Xinjiang Academy of Architectural Science (Limited Liability Company), Urumqi, China; 4 Department of Geotechnical Engineering, Tongji University, Shanghai, China; Sapienza University of Rome: Universita degli Studi di Roma La Sapienza, ITALY

## Abstract

The mechanical properties of deep rock masses are significantly influenced by temperature and other factors. The effect of temperature on the strength of deep rock masses will pose a serious challenge to deep resource exploitation and engineering construction. In this paper, the thermal-mechanical coupling calculation model is established by particle flow code (PFC^2D^) to study the uniaxial compression response of rock masses with microcracks after temperature load. The strength of failure, microcracks, and strain was analyzed. The results show that: (i) When the soft rock thickness ratio *Hs/H* < 0.5, the displacement caused by the applied temperature is concentrated at the structural plane, and the contact force is concentrated at the end of the initial microcrack. When *Hs/H* ≥ 0.5, the displacement caused by the applied temperature is concentrated on both sides of the initial microcrack, and the contact force is concentrated in the hard rock area. (ii) The number of microcracks decreases with the increase of soft rock thickness under different working conditions. When the soft rock thickness ratio *Hs/H* < 0.5, the relationship curve between the number of microcracks and the vertical strain shows two stages of change. When *Hs/H* ≥ 0.5, the relationship curve between the number of cracks and the vertical strain changes shows three stages of change. (iii) When the soft rock thickness ratio *Hs/H* < 0.5, the failure strength decreases with the increase of soft rock thickness ratio at *T* = 100°C and 200°C. When *T* = 300°C and 400°C, the failure strength decreased first and then increased. When *Hs/H* ≥ 0.5, the failure strength increases with the increase of soft rock thickness at *T* = 200°C, 300°C, and 400°C. At *T* = 100°C, the failure strength decreases with the increase of soft rock thickness.

## Introduction

As shallow-crust resources are increasingly depleted, the exploitation of deep resources and the construction of engineering projects are increasing. As the depth of resource occurrence increases, the increasingly prominent problem of high ground temperatures will pose severe challenges to deep geotechnical engineering practices such as deep resource mining. Exploring the influence of temperature on the mechanical properties and failure characteristics of deep rock masses will be an important way to address such questions. The influence of temperature on the mechanical properties of rock masses has been revealed by relevant studies [1–4]. Mutlutürk et al. [5] established a mathematical model to describe the loss of rock mass integrity after repeated freezing and thawing. In the study of granite subjected to freezing and thawing, scholars have studied and analyzed the mechanical properties and failure characteristics of granite after freezing and thawing in terms of uniaxial compressive strength, tensile strength, and water absorption [6]. At the same time, some scholars used acoustic emission and electron microscopic scanning techniques to study the development and expansion of thermal microcracks and the expansion of non-uniform crystals in granites in different regions after being affected by temperature. The results show that the crack growth is affected by the grain shape of the mineral and the thermal crack distribution. Increasing the temperature reduces the stress threshold of crack occurrence and failure, and prolongs the stable propagation time of cracks [7, 8]. To analyze the influence of the thermal conductivity of evaporites on different rock masses, Pauselli, C et al. [9] used a two-probe method to predict the thermal conductivity of evaporites. At the same time, various hybrid models have been used to verify the predictions, and the excellent agreement of the results proves the correctness of the adopted model. Some studies have analyzed the effect of temperature on the physical and mechanical properties of sandstone from the aspects of void characteristics, elastic modulus, and uniaxial compressive strength [10, 11]. Some scholars have studied and analyzed the fracture toughness of rock mass in Mode I and Mode II under different temperature conditions, revealing the effect of temperature on the two modes [12, 13]. Song et al. [14] studied and analyzed interlayered marbles with dip angles of 0°, 30°, 45°, 60°, and 90°. The results show that marble samples with interlacing fail in the split mode, and that interlacing with different inclination angles has a weakening effect on the rock strength. Chang et al. [15] used acoustic emission tests, failure modes, and load-displacement responses to study the failure mechanism of unreinforced and reinforced layered rocks with different layer dips. The results show that the failure mode changes from a split hard layer to a shear between weak layers with increasing interlayer inclination for both unreinforced and reinforced specimens. For the effect of temperature on the physical and mechanical properties of rock mass, numerous scholars have conducted a lot of analysis and research from different perspectives [4, 16–18]. Qiao et al. [19] studied and analyzed the internal relationship between mechanical properties and acoustic emission energy under temperature effect. Based on the theory of energy dissipation and release, the self-excitation and self-inhibition models of energy are established. Trippetta F et al. [20] ascent to a larger geophysical survey. The density, porosity, Vp, Vs, seismic anisotropy and permeability of evaporite under an effective confining pressure of 0 ~ 100 MPa were studied. Chen et al. [21] used the elemental method to study the failure process of interlayer rock masses with different dip angles. At the same time, the expression of interfacial bond stress is established based on the fracture theory of layered rock mass. Some scholars have carried out research and analysis on the effect of temperature on the friction angle, cohesion, and tensile strength of rock mass [22–25]. Tan et al. [26] studied and analyzed the mechanical properties of granite after freezing and thawing in terms of strength, deformation characteristics, elastic modulus, cohesive strength, and internal friction angle. Mohsen et al. [27] explored the factors that may affect the initiation of cracks and the possible initiation mechanism. Based on the particle model, the effects of particle size distribution, particle size, and heterogeneity of different mineral particles on fracture occurrence were studied. Han et al. [28] used PFC^2D^ to establish a numerical model including the variable thickness and undulation of ISWZ, and studied and analyzed the characteristics of the interlayer shear weak zone (ISWZ) of layered rock mass through the direct shear test. Tao et al. [29] proposed a novel thermodynamic framework to describe the sequence of continuous blasting loads, instantaneous unloading, and air cooling for dynamic excavation of prestressed geothermal deposits. At the same time, to capture the behavior of complex rock masses on different time scales, a new judgment basis is proposed to determine the exact source, nature and scope of excavation damage. Yin et al. [30] proposed an effective measurement method for high-temperature rock dynamic mode I fracture toughness, and combined the discrete element method (DEM) to study and analyze the mechanical properties of the rock mass. To analyze the influence of seismic period on the physical properties of Evaporites, Trippetta F et al. [31] evaluated the physical and mechanical properties of Evaporites by using static (Es, νs) and dynamic (Ed, νd) uniaxial compressive strength (UCS), Young’s modulus and Poisson’s ratio. Liu et al. [32] proposed a current prediction model of uniaxial compressive strength against freezing and thawing based on elastic-plastic theory and fatigue failure mechanics and considering the actual stress distribution of rock after freezing and thawing. The predictive model is used to determine the long-term uniaxial compressive strength loss of rocks after freezing and thawing. Tao et al. [33] established a new fully coupled thermo-hydraulic-chemical model for consolidation backfill citing fundamental conservation equations. The model takes into account the thermal pressure effect due to the thermal expansion of pore water, which is missing from the existing backfilling models. Zhang et al. [34] studied rock samples in terms of mineral composition, chromaticity, mass, thermal conductivity, and P-wave velocity. The failure mode of the rock with a single dip angle initial crack is revealed under the action of high temperature. It was also found that pre-existing cracks caused the failure mode of tabular granite specimens to shift from intermediate splitting to *Y*-shear failure.

Many scholars have studied the influence of temperature on the strength and failure mode of rock mass in different aspects, and the research results have provided a certain reference for engineering construction. However, the existing research mainly focuses on the effect of temperature on the mechanical characteristics of rock mass under the condition of a single factor. For deep rock mass, its strength is affected by high temperature and various geological factors. In the existing research, there are few research results on the effect of high temperature on the mechanical characteristics of interbedded rock mass with microcracks. Make its lack of reference in engineering construction. Therefore, it is very necessary to carry out related research to make up for the lack of research on the deformation and damage of interbedded rock mass with microcracks at high temperatures. Based on discrete element (DEM) software PFC, the discontinuity and joints and other structural characteristics of the rock mass can be well simulated. In this paper, the discrete element method is used to establish a thermal-mechanical coupled numerical calculation model to study and analyze the influence of high temperature on the mechanical properties of interbedded rock masses with microcracks.

## Theory of discrete element thermal computation

### Governing equations

In the thermal calculation module of the particle flow code (PFC^2D^), the calculation method of continuum heat conduction is adopted. When deriving the heat conduction equation, it is assumed that the effect of strain change on temperature is negligible. Then the formula for heat conduction in the continuum is [35]:

$$-\frac{\partial q_{i}}{\partial x_{i}}+q_{v}=\rho C_{v}\frac{\partial T}{\partial t}$$ (1)

where, *q*_*i*_ is the heat-flux vector (W/m^2^). *q*_*v*_ is the volumetric heat-source intensity or power density(W/m^3^). *ρ* is the mass density(kg/m^3^). *C*_*v*_ is the specific heat at constant volume(J/kg). *T* is the calculation temperature. Fourier’s law for a continuum defines the relation between the heat-flux vector and the temperature gradient as:

$$q_{i}=-k_{ij}\frac{\partial T}{\partial x_{j}}$$ (2)

where, *k*_*ij*_ is the thermal-conductivity tensor.

### Thermal contact properties

In the particle flow code (PFC^2D^), the linear parallel bond model takes into account the thermal properties of bond contact between particles. When considering the thermal effect, it is assumed that the temperature change is Δ*T*, and the amount of radius change of the granular material is [35]:

ΔR=αRΔT (3)

where, *α* is the coefficient of linear thermal expansion associated with the particle, and *R* is the initial radius of granular material. At the same time, the calculation of mechanical contact mechanics considers thermal contact, assuming that the particle material has only normal bonding force $\Delta \bar{F_{n}}$ and is affected by temperature. Assuming that the bond material is linear isotropic expansion, and the effective bond length between particles is $\bar{L}$. The variation of the normal bond force component is [35]:

$$\Delta \bar{F_{n}}=-\bar{k_{n}}A(\alpha \bar{L}\Delta T)$$ (4)

where, $\bar{k_{n}}$ is the bond’s normal stiffness. *A* is the area of the bond cross-section(m^2^). *α* is the expansion coefficient of the bond material. Δ*T* is the temperature increment.

## Temperature loading and verification

### Model parameter selection

**Fig 1:**
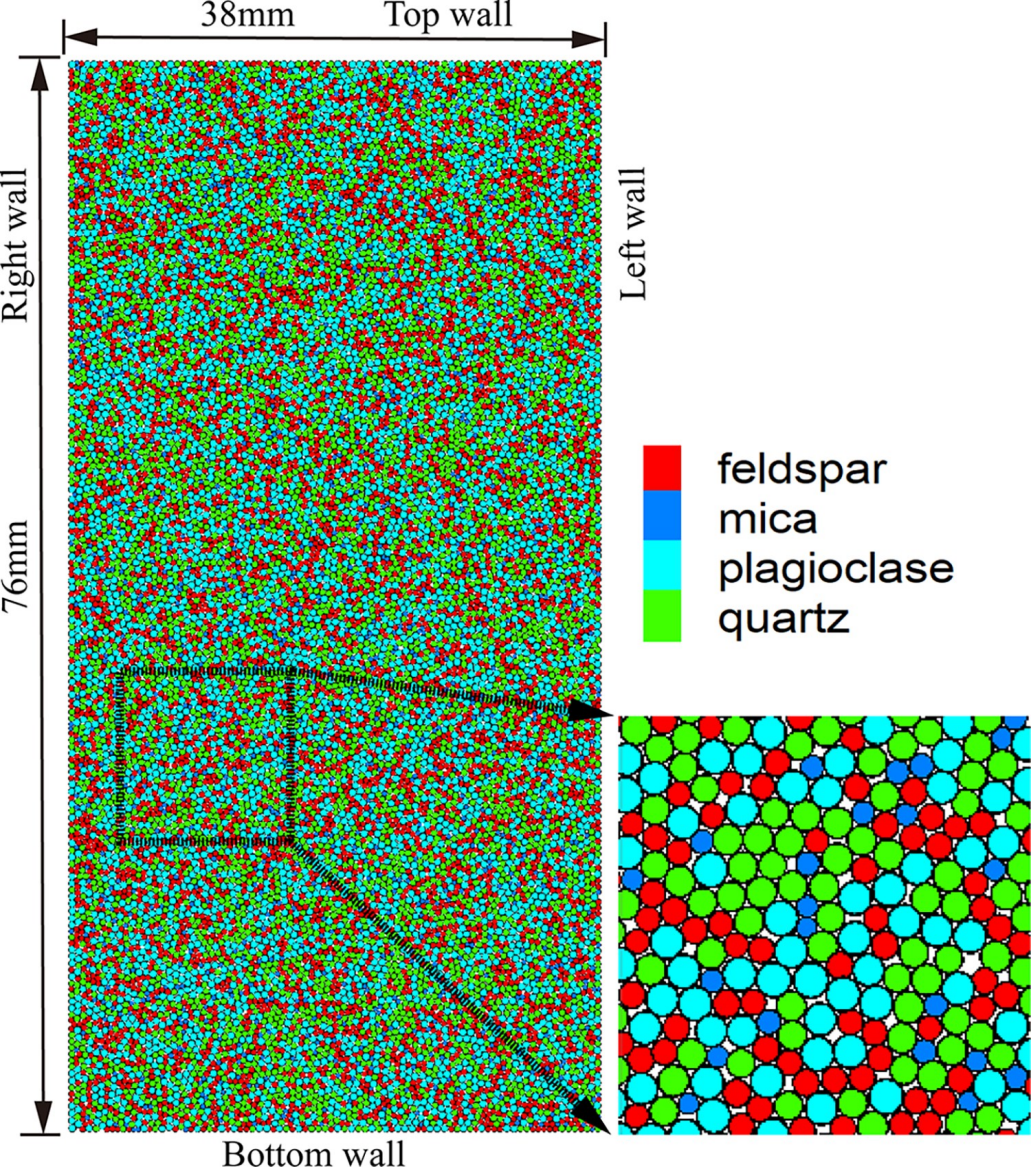
Thermal-mechanical coupling verification calculation model.

In this paper, the uniaxial compression model established is shown in Fig 1. The size of the model is 76mm×38mm and consists of 15754 particles. The calculation model adopts the linear parallel bonding model. The sample particles were divided into four groups based on the mineral species, namely quartz, feldspar, plagioclase, and mica. The thermal expansion coefficients of different minerals are shown in Table 1 [36]. The thermal resistance of the activated heat pipe is calculated according to the thermal conductivity (Eq 5), and the thermal conductivity *K* is 3.5Wm^-1^K^-1^. The calculated model thermodynamic parameter specific heat is 1015J kg^-1^K^-1^ [37].

**Table 1:** The thermal expansion coefficient of different minerals.

Mineral name	Thermal expansion coefficient (K^-1^)
Quartz	24.3×10^−6^
feldspar	8.7×10^−6^
Plagioclase	14.1×10^−6^
Mica	3.0×10^−6^


$$\eta =\frac{1}{2K}(\frac{1-\phi }{\sum_{N_{b}}V_{b}})\sum_{N_{p}}l_{p}$$ (5)

where, *ϕ* is the porosity. *V*_*b*_ is the particle volume. *l*_*p*_ is the contact length of the heat pipe. *N*_*b*_ and *N*_*p*_ are the number of particles and the number of contact heat pipes, respectively.

### Boundary conditions and temperature loading methods

**Fig 2:**
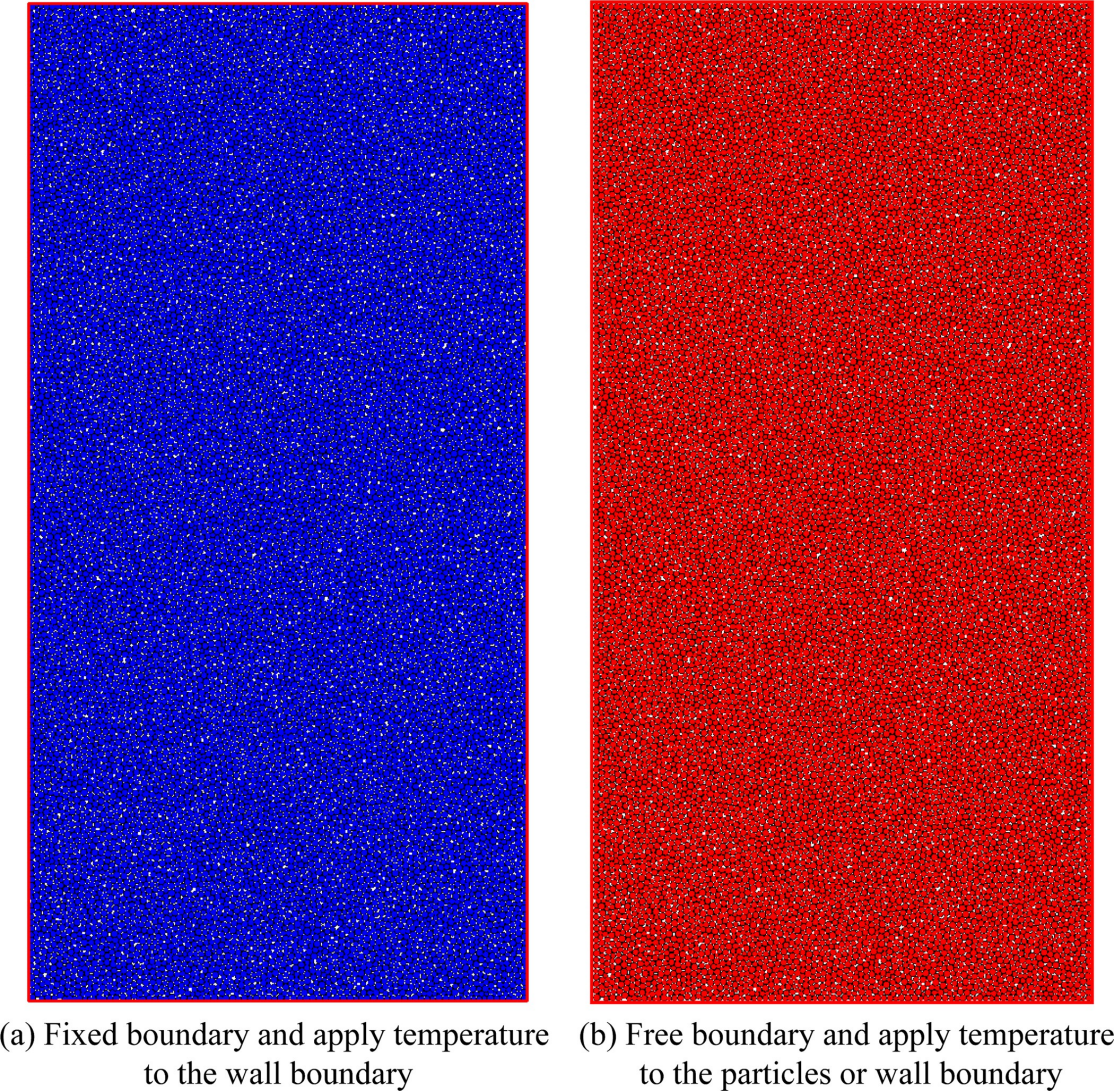
Temperature boundary condition setting.

In the PFC^2D^ thermal calculation module, the model boundary can be fixed or free. When applying temperature to the sample, the model boundary wall or boundary particles can be used as the heat source to apply the temperature, or the temperature can be directly applied to the entire sample. The boundary conditions and temperature application methods are shown in Fig 2.

**Fig 3:**
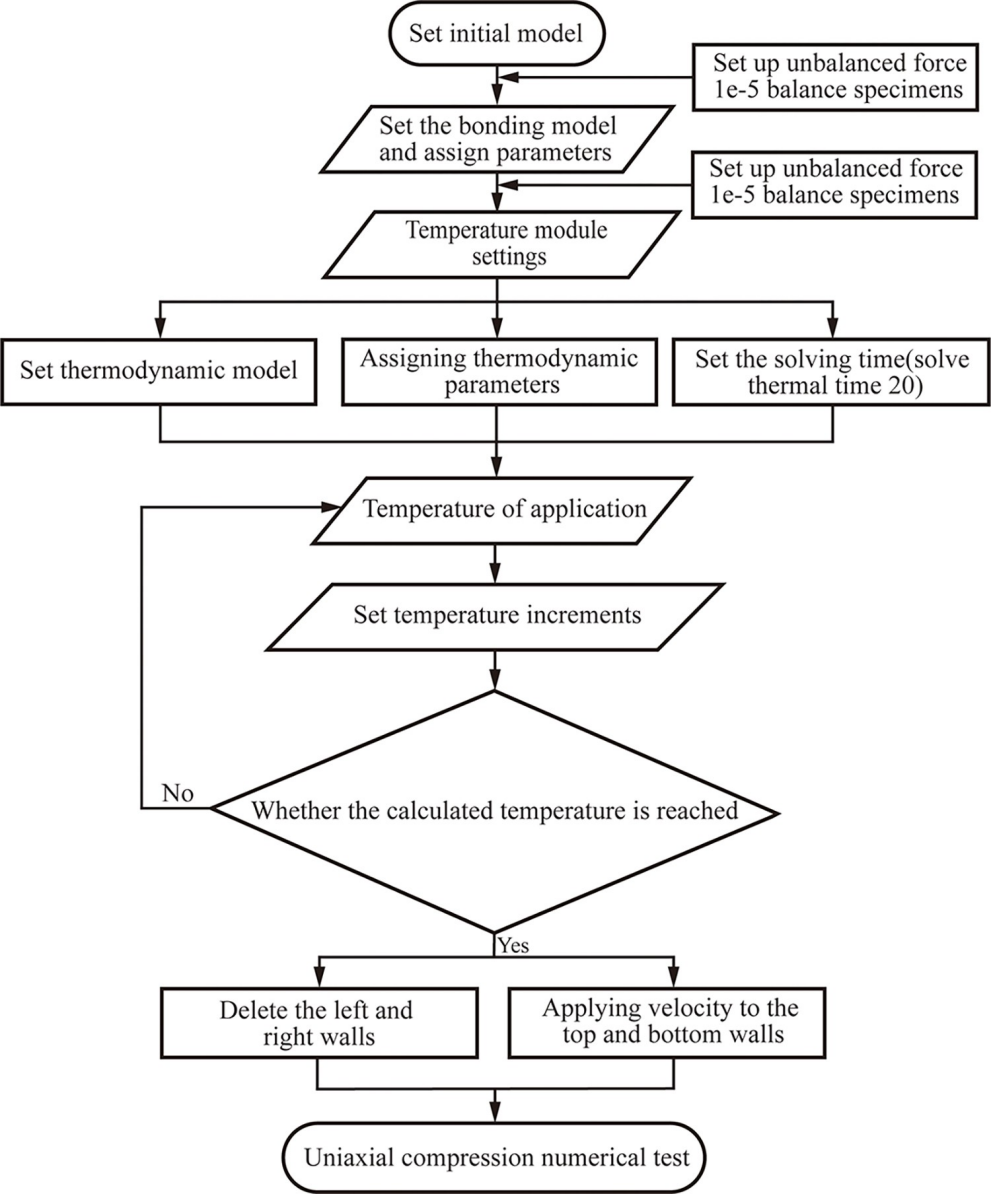
Numerical calculation process.

To reduce the influence of the boundary on the thermal expansion of the particles, free boundary conditions are used in this paper. At the same time, the wall is used as the heat source to apply the temperature to the sample. The initial temperature of the sample and the wall is 20°C. The wall temperature is increased after equilibration for each temperature calculation. The temperature increment is 10°C, and the calculation solution time is the 20s (solve thermal time 20). The numerical procedure is mainly divided into four stages: building the initial model, setting the contact model, applying the temperature, and uniaxial compression, as shown in Fig 3.

### Analysis of model validation results

**Fig 4:**
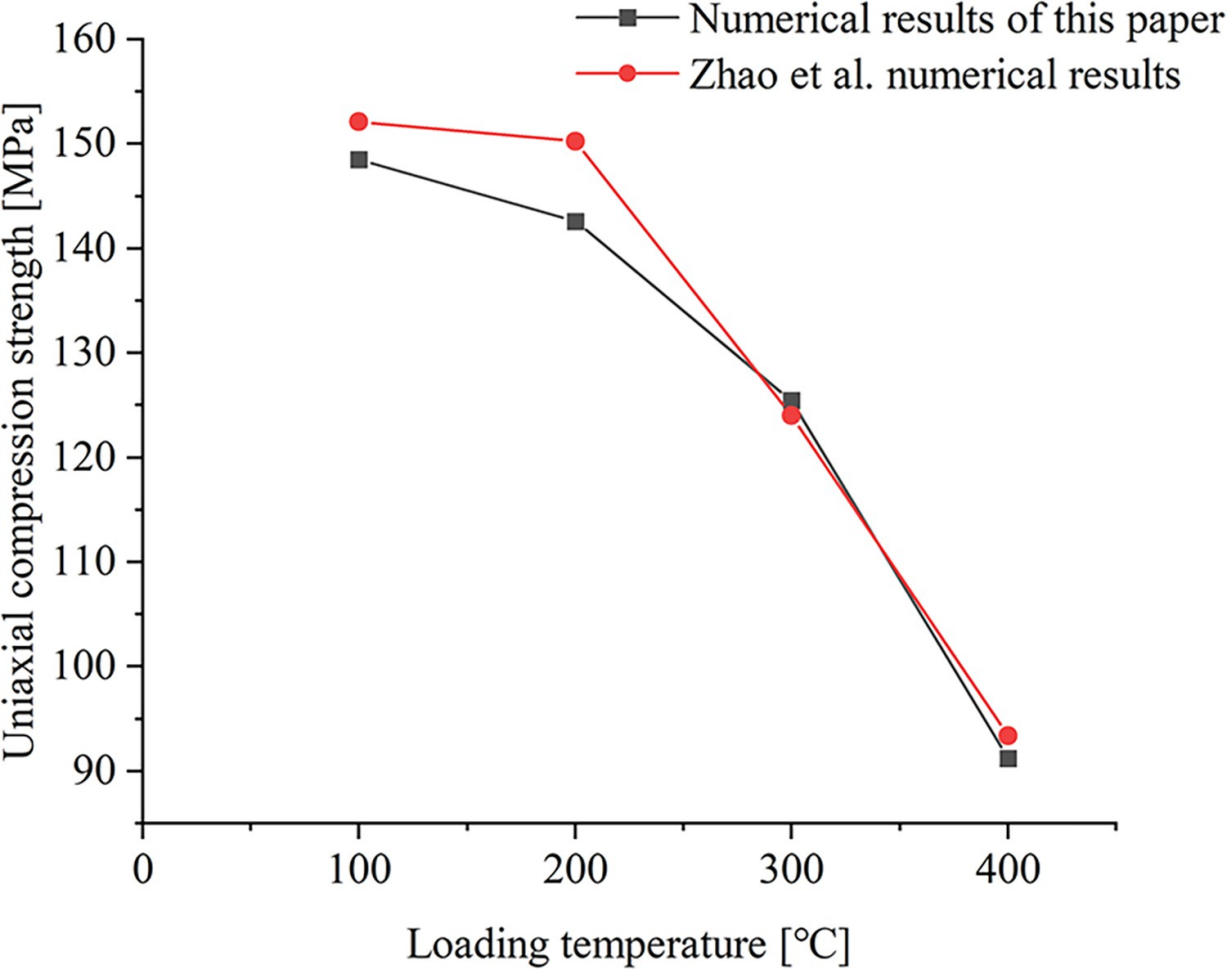
Comparison of numerical simulation in this paper with the numerical results of Zhao et al. [38].

The numerical validation model was established by using the above model building method and temperature application method. The numerical procedure is shown in Fig 3. Fig 4 shows the failure strength of the sample at different temperatures obtained from the simulation calculations. When the model size, particle grouping, and rock mechanical properties are consistent, the resulting error percentages are 2.4%, 5%, 1.2%, and 2.3%, respectively. The error in the calculation is small. And the uniaxial strength change trend obtained in this paper is consistent with the intensity change trend obtained by Zhao et al. [38], which proves the rationality of the temperature loading method adopted in this paper.

## Working condition setting and analysis of temperature loading results

The strength of soft-hard interbedded rock masses with microcracks is not only affected by its characteristics, but the temperature is also an important factor. To explore the influence of temperature on mechanical characteristics of soft-hard interbedded rock masses with microcracks, the temperature loading is carried out on the rock mass samples of different thicknesses and the mechanical characteristics of the rock mass samples are analyzed.

### Working condition setting

In order to analyze the strength characteristics of soft hard interbedded rock mass with microcracks under different temperature conditions. In this paper, sandstone and limestone are studied. In this paper, the microscopic parameters of sandstone used by Yuan et al. [39] and limestone used by Xie et al. [40] were used as numerical calculation parameters. Yuan and Xie et al. conducted multiple sets of physical experiments to obtain the macroscopic parameters of the rock. At the same time, the PFC software was used to calibrate the microscopic parameters based on the macroscopic parameters such as uniaxial compression strength, Young’s modulus and Poisson’s ratio. The microscopic parameters (Table 2) used in this paper were obtained through a large number of calibration tests, and the reliability of the parameters was also proved. The thermal conductivity *K*, required for the model calculations is 3.5Wm^-1^K^-1^, and the specific heat is 1015J kg^-1^K^-1^.

**Table 2:** Calculation of required rock microscopic parameters.

Parameters (unit)	Value	
	Sandstone	Limestone
Bond effective modulus (GPa)	42.0	2.5
Bond stiffness ratio	1.0	1.8
Bond tensile strength (MPa)	30.0	10.0
Bond cohesion (MPa)	350.0	5.0
Bond friction angle (°)	65	10

**Fig 5:**
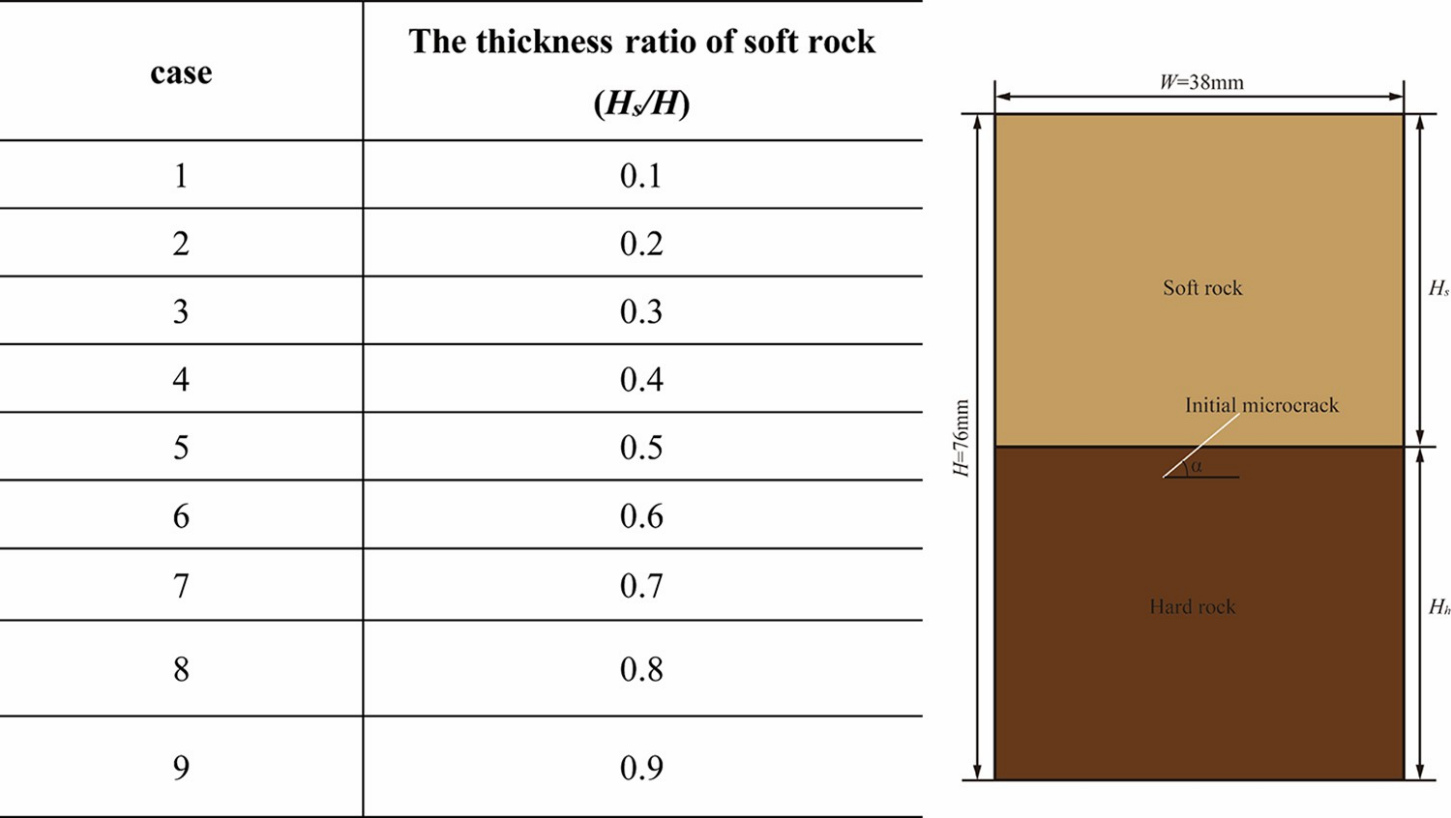
Numerical calculation of working conditions.

To analyze the mechanical properties of soft-hard interbedded rock mass with microcracks under different temperature conditions, the size of the sample model is 76mm×38mm. The linear parallel bonding model was used for the calculation. The different working conditions are listed in Fig 5. The numerical experiments were carried out at 100°C, 200°C, 300°C, and 400°C. Microcracks have an important influence on the mechanical properties of rocks. Some scholars have analyzed the mechanical properties of rock with different crack dip angles (30°, 45° and 60°, etc.) and obtained relevant conclusions [41]. Based on the research of scholars, this paper selects one of the typical crack inclination angles. The initial microcrack inclination angle is set to 30°, and the microscopic parameters are shown in Table 3. The boundary conditions and temperatures are applied in the same way as in the previous model validation stage. The boundary wall is used as the heat source to apply the temperature to the sample. The wall temperatures at the top and bottom of the model were kept constant [42]. When the boundary temperature reaches the calculated temperature, the next temperature application is carried out, and the temperature increment is 10°C. When the boundary temperature reaches the final calculated temperature, the temperature loading is stopped and a numerical test of uniaxial compression is performed.

**Table 3:** Microcrack microscopic parameters.

Parameters (unit)	Value
Normal stiffness per unit area (N/m^2^)	2e9
Shear stiffness per unit area (N/m^2^)	2e9
Friction coefficient	0.35

### Analysis of displacement during temperature application

**Fig 6:**
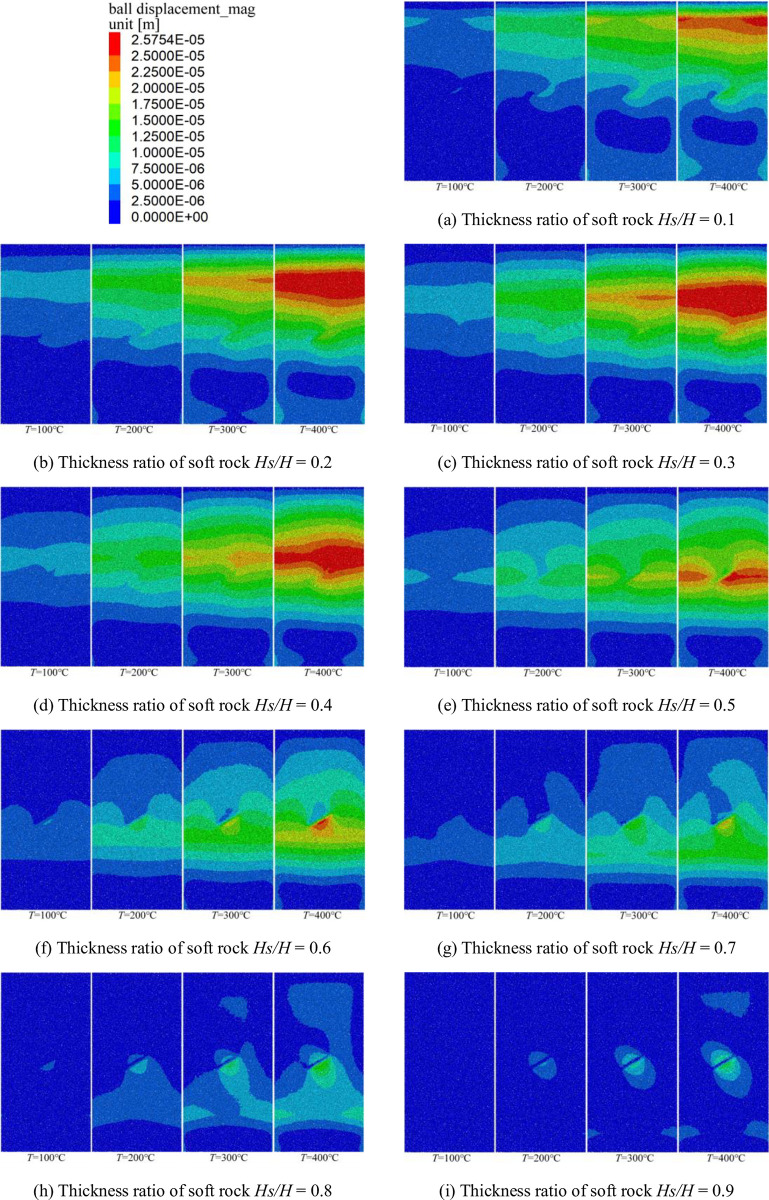
Displacement caused by temperature under different working conditions. (a) Thickness ratio of soft rock *Hs/H* = 0.1, (b) Thickness ratio of soft rock *Hs/H* = 0.2, (c) Thickness ratio of soft rock *Hs/H* = 0.3, (d) Thickness ratio of soft rock *Hs/H* = 0.4, (e) Thickness ratio of soft rock *Hs/H* = 0.5, (f) Thickness ratio of soft rock *Hs/H* = 0.6, (g) Thickness ratio of soft rock *Hs/H* = 0.7, (h) Thickness ratio of soft rock *Hs/H* = 0.8, and (i) Thickness ratio of soft rock *Hs/H* = 0.9.

Because the sample particles have different thermal expansion coefficients. Therefore, when the temperature is applied to the sample, there will be different degrees of thermal expansion, resulting in a certain difference in the displacement of the sample. The displacement generated by temperature under different working conditions is shown in Fig 6.

It can be seen from Fig 6 that the large displacement area gradually expands with increasing temperature for the same soft rock thickness ratio. When the soft rock thickness ratio *Hs/H* < 0.3, the large displacement region generated by the applied temperature expands continuously with the increase of the soft rock thickness ratio. However, when the soft rock thickness ratio *Hs/H* > 0.3, the large-displacement influence zone caused by the application of temperature gradually decreases with the increase of the soft rock thickness ratio. The extent of the large displacement region reaches its minimum when the soft rock thickness ratio is *Hs/H* = 0.9. When the soft rock thickness ratio *Hs/H* < 0.5 (the soft rock thickness is less than the hard rock thickness), the displacement of the sample by applied temperature is mainly concentrated at the structural surface of the soft rock and the hard rocks. When the thickness ratio of soft rock is *Hs/H* > 0.5 (the thickness of soft rock is greater than that of hard rock), the large displacement caused by the applied temperature is mainly concentrated near the initial microcrack, and the displacement value is small. However, the displacement is smaller at the structural plane of soft and hard rock. When the thickness of hard rock is large, the structural plane is the main factor affecting the displacement distribution. When the soft rock thickness is larger, the displacement distribution is affected by the initial microcracks, but the structural face has little influence on the displacement distribution.

**Fig 7:**
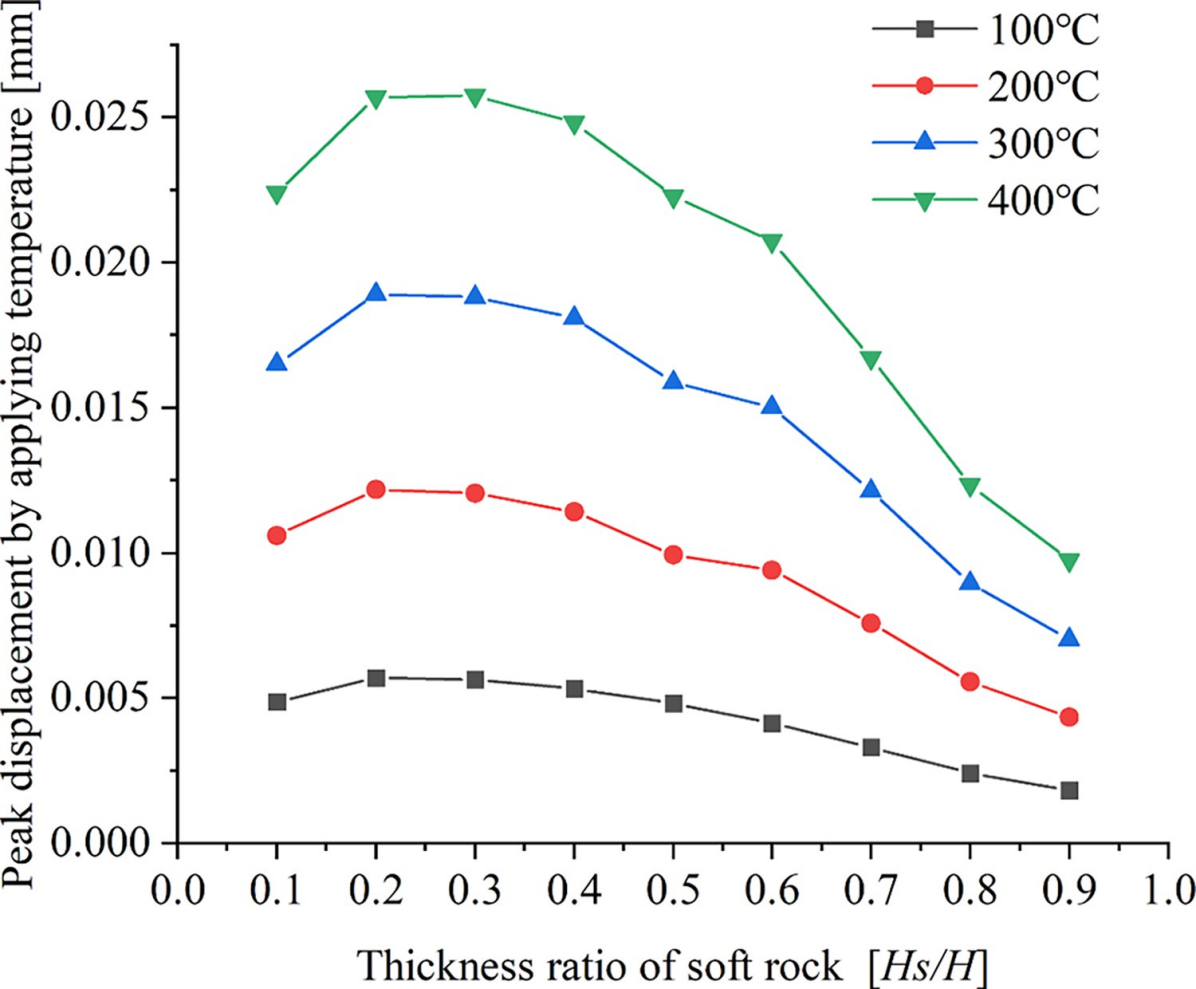
Relationship curve between peak displacement and thickness ratio of soft rock.

Fig 7 shows the relationship between the peak displacement generated by the applied temperature and the soft rock thickness ratio under different working conditions.

As can be seen from Fig 7, the relationship between the peak displacement and the soft rock thickness ratio is a quadratic function. The peak displacement first increases and then decreases with increasing soft-rock thickness ratio, with a more significant trend at higher temperatures. Under different temperature conditions, the peak displacement has the smallest value when the soft rock thickness ratio is *Hs/H* = 0.9, and the peak displacement difference is the smallest for all four temperatures. This result is consistent with the variability in the range of large displacement regions presented in Fig 6. When the thickness ratio of soft rock is *Hs/H* = 0.9, the influence range of displacement is minimal. The peak displacement has the largest value at four temperatures for the soft rock thickness ratio *Hs/H* = 0.3. During the temperature application phase, the influence of temperature on the displacement of rock-mass samples gradually weakened as the thickness of the soft rock increases.

### Analysis of contact force generated in the stage of applying temperature

**Fig 8:**
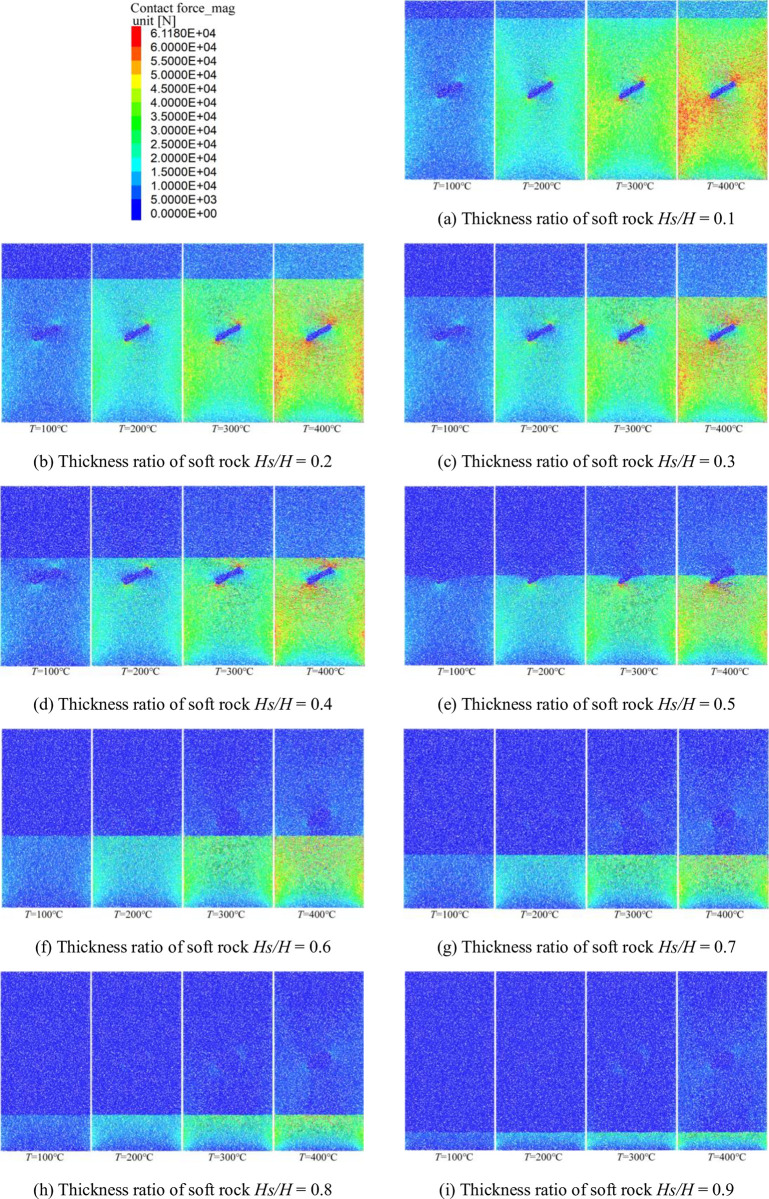
Contact force generated by applying temperature under different working conditions. (a) Thickness ratio of soft rock *Hs/H* = 0.1, (b) Thickness ratio of soft rock *Hs/H* = 0.2, (c) Thickness ratio of soft rock *Hs/H* = 0.3, (d) Thickness ratio of soft rock *Hs/H* = 0.4, (e) Thickness ratio of soft rock *Hs/H* = 0.5, (f) Thickness ratio of soft rock *Hs/H* = 0.6, (g) Thickness ratio of soft rock *Hs/H* = 0.7, (h) Thickness ratio of soft rock *Hs/H* = 0.8, and (i) Thickness ratio of soft rock *Hs/H* = 0.9.

In the process of temperature, the thermal expansion of the sample particles changes the contact characteristics of the sample particles. This results in a change in the contact force between the particles. Fig 8 shows the distribution of contact forces for a rock-mass sample after temperature loading is completed for different operating conditions.

As can be seen from Fig 8, the contact force between the sample particles is significantly affected by temperature. Under the same soft-rock thickness ratio, the contact force between particles increases gradually with the increase of the applied temperature, and the range of influence of the contact force gradually expands. When the thickness ratio of soft rock is *Hs/H* < 0.5 (the thickness of soft rock is less than that of hard rock), the contact force generated by the applied temperature is mainly concentrated in the hard rock area, and the contact force is larger at the end of the initial microcrack. When the soft rock thickness ratio *Hs/H* > 0.5 (the soft rock thickness is greater than the hard rock thickness), the contact force is mainly concentrated in the hard rock area. The contact force generated near the initial microcracks is smaller in the soft rock region. Overall, it can be seen that when the thickness of the hard rock is large and the initial micro-cracks are in the hard rock region, the temperature has a large influence on the contact force between the sample particles. When the initial microcracks are in the soft rock region, the contact force between the particles of the rock-mass sample is less affected by the temperature.

**Fig 9:**
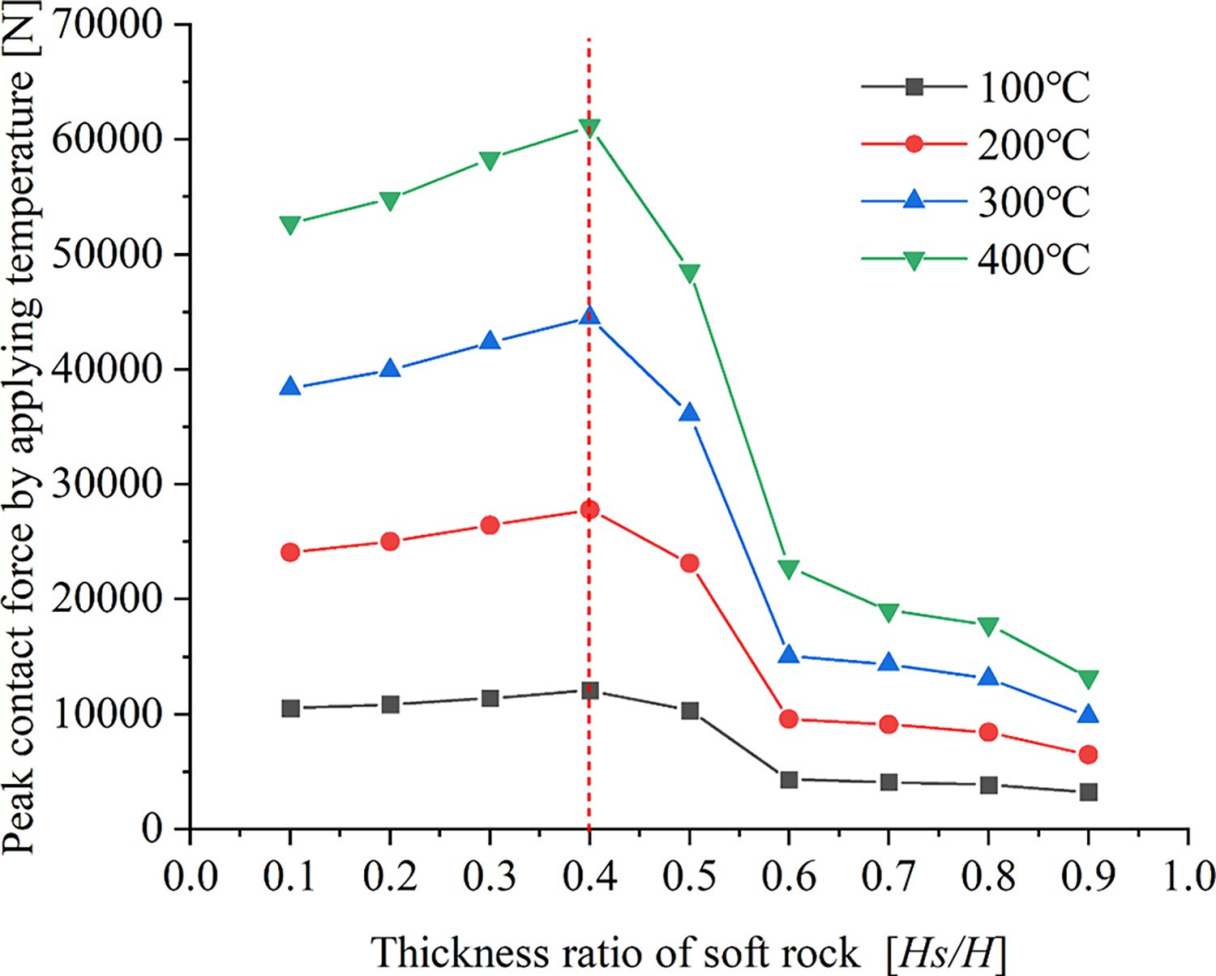
Relationship curve between peak contact force and thickness ratio of soft rock.

The resulting relation between the peak contact force and the soft rock thickness ratio is shown in Fig 9 for different working conditions.

It can be seen from Fig 9 that the peak contact force generated is greatly affected by the temperature at different conditions, showing mainly two phases of variation. When the soft rock thickness ratio *Hs/H* < 0.4, the peak contact force gradually increased with the increase of the soft rock thickness ratio, and the higher the temperature, the more significant the increasing trend was. At the same time, Fig 8 shows that when the thickness ratio of soft rock is in the range of 0.1 to 0.4, the contact force is large and has a large range of effects. When the soft rock thickness ratio *Hs/H* > 0.4, the greater the soft rock thickness, the smaller the peak contact force. When the thickness ratio of soft rock is *Hs/H* = 0.4~0.6 (initial microcracks penetrate the soft rock and hard rock structural plane), the trend of decreasing peak contact force is larger for the four temperature conditions. It shows that the initial microcrack has a significant impact on the peak contact force when the initial microcrack penetrates the structural surface. When the soft rock thickness ratio is *Hs/H* = 0.6~0.9 (the microcracks are located in the soft rock area), the peak contact force gradually decreases with the increase of the soft rock thickness, and the decreasing trend is relatively gentle. According to Fig 8, the influence range of the contact force gradually decreases in the range of *Hs/H* = 0.6~0.9, and it is mostly in the hard rock area. Comprehensive analysis shows that the contact force of hard rock particles is significantly affected by temperature. The location of the initial microcrack will have a certain influence on the magnitude and distribution of the contact force.

## Analysis of the effect of temperature load on the mechanical properties of rock mass

To analyze the influence of temperature on the mechanical properties of the rock masses. In this paper, temperature loading is carried out, and uniaxial compression numerical test is carried out. The microcrack development, vertical strain and uniaxial strength were analyzed.

### Microcrack analysis

**Fig 10:**
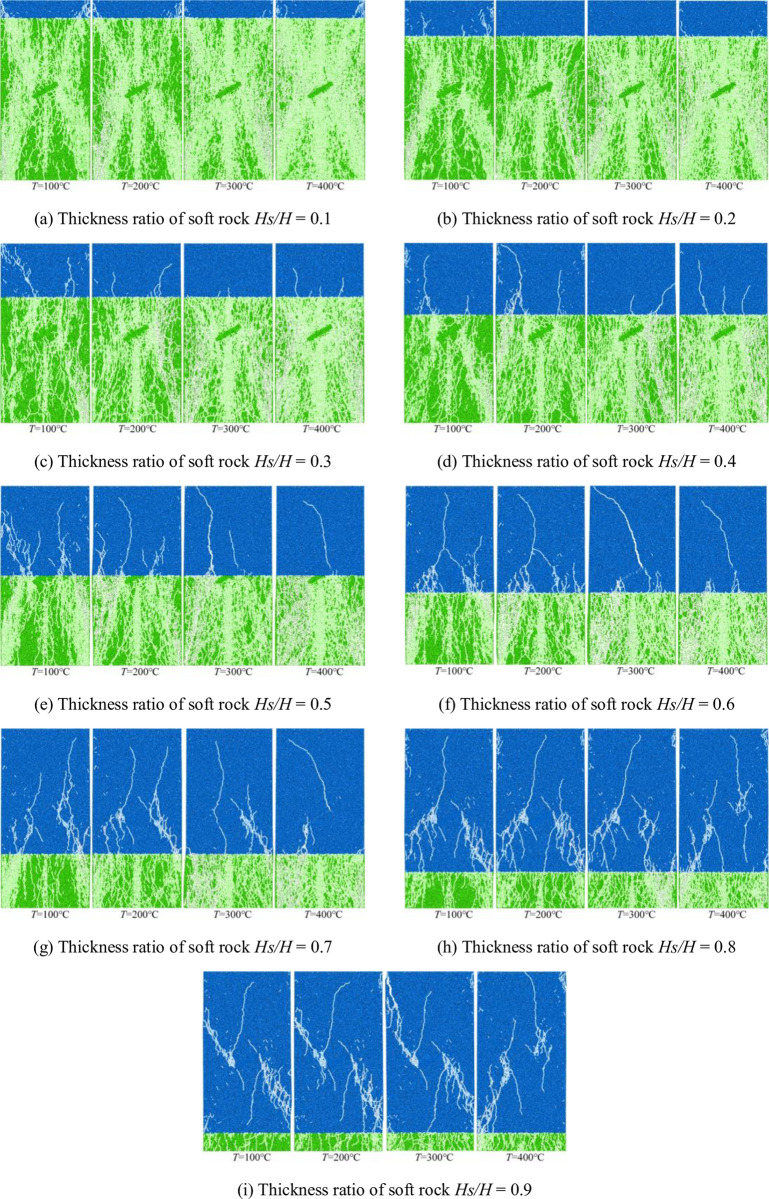
Microcrack development under different working conditions. (a) Thickness ratio of soft rock *Hs/H* = 0.1, (b) Thickness ratio of soft rock *Hs/H* = 0.2, (c) Thickness ratio of soft rock *Hs/H* = 0.3, (d) Thickness ratio of soft rock *Hs/H* = 0.4, (e) Thickness ratio of soft rock *Hs/H* = 0.5, (f) Thickness ratio of soft rock *Hs/H* = 0.6, (g) Thickness ratio of soft rock *Hs/H* = 0.7, (h) Thickness ratio of soft rock *Hs/H* = 0.8, and (i) Thickness ratio of soft rock *Hs/H* = 0.9.

The results of the previous analysis show that temperature has a strong influence on the bonding properties between particles. Due to the change in the cohesive properties of the sample particles, the development of microcracks under uniaxial compression is significantly different. The development of microcracks under uniaxial compression is shown in Fig 10.

It can be seen from Fig 10 that under the condition of soft rock thickness ratio *Hs/H* < 0.5 (soft rock thickness is less than hard rock thickness), the development of microcracks is mainly concentrated in the hard rock area. At the same soft-rock thickness ratio, microcracks in hard-rock regions are significantly affected by temperature. The higher the temperature, the more significant the development of micro-cracks. And the rock mass in the hard rock area is relatively fragmented. Smaller microcracks are generated in the soft rock region, and the microcracks develop through the structure from the hard rock area to the soft rock area. When the thickness ratio of soft rock is *Hs/H* > 0.5 (the thickness of soft rock is greater than that of hard rock), the development of microcracks is significantly affected by the initial microcracks in the soft rock region. The microcracks generated in the compression phase gradually develop from the ends of the initial microcracks, and some of the microcracks extended into regions of hard rock. The hard rock area is greatly affected by temperature. The higher the temperature, the more microcracks are generated in the hard rock area. During the uniaxial compression stage, the initial microcracks have little effect on the development of microcracks when they are located in the hard rock region. However, when the initial microcrack is located in the soft rock region, it has a great influence on the development of the microcrack.

**Fig 11:**
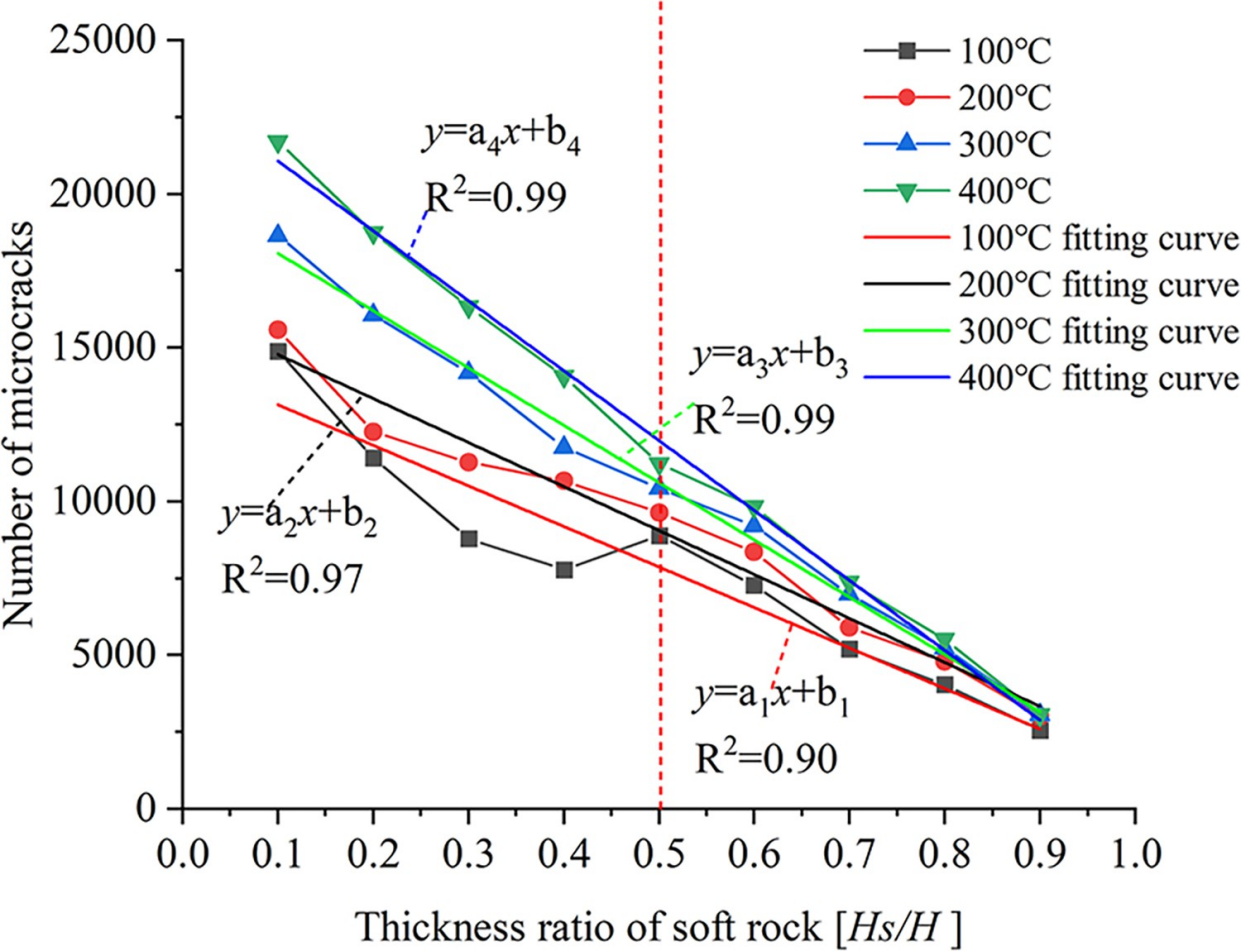
The relationship curve between the number of microcracks and the thickness ratio of soft rock under different temperature conditions.

Fig 11 shows the relationship between the thickness ratio of soft rock and the microcracks generated at the compression stage under four temperature conditions.

As can be seen from Fig 11, the number of microcracks gradually decreases with increasing soft rock thickness. Under the condition of soft rock thickness ratio *Hs/H* < 0.5 (soft rock thickness is less than hard rock thickness), the number of microcracks decreases first and then increases with the increase of soft rock thickness when loading temperature *T* = 100°C, and the increasing trend is relatively flat. When the loading temperature is *T* = 200°C, 300°C and 400°C, the number of microcracks gradually decreases with the increase of soft rock thickness, and the decreasing trend is more significant with higher temperatures. When the soft rock thickness ratio *Hs/H* > 0.5, the larger the soft rock thickness, the less the number of microcracks generated in the compression stage. When the soft rock thickness ratio *Hs/H* = 0.9, there is a minimum in the number of microcracks for the four temperatures. The fitting curve of the number of microcracks and the thickness ratio of soft rock under four temperature conditions is shown in Fig 11. The resulting fitting formula is:

$$y=a_{i}x+b_{i}$$ (6)

where, *a*_i_ and *b*_i_ is fitting parameters, and the parameter values are shown in Table 4.

**Table 4:** Curve fitting parameters for the relationship between microcracks and soft rock thickness ratio.

Temperature (°C)	a_i_(i = 1,2,3,4)	b_i_(i = 1,2,3,4)
100	-13187.33	14463.44
200	-14303.33	16208.89
300	-18640.83	19935.08
400	-22739.33	23352.44

The obtained fitting curve and its parameters can be used to approximate the number of microcracks under other temperature conditions, and the degree of fragmentation of rock samples can be judged. When the thickness of the soft rock is smaller than that of the hard rock, the microcracks develop significantly at all four temperature conditions, and the failure of the sample is dominated by the hard rock. When the thickness of soft rock is larger than that of hard rock, the number of microcracks generated during the compression stage gradually decreases under the four temperature conditions, and the failure of the sample is dominated by the soft rock.

### Vertical strain analysis

**Fig 12:**
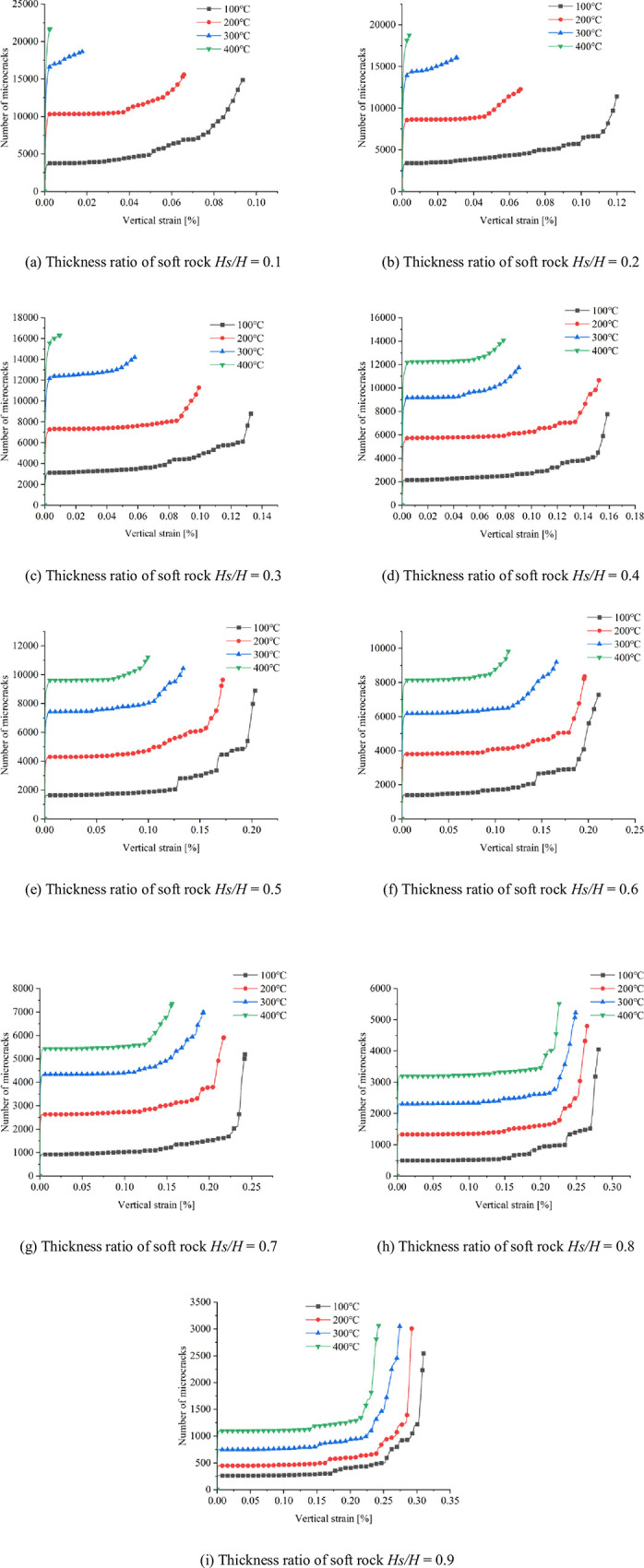
Relationship curve between the number of microcracks and vertical strain under different working conditions. (a) Thickness ratio of soft rock *Hs/H* = 0.1, (b) Thickness ratio of soft rock *Hs/H* = 0.2, (c) Thickness ratio of soft rock *Hs/H* = 0.3, (d) Thickness ratio of soft rock *Hs/H* = 0.4, (e) Thickness ratio of soft rock *Hs/H* = 0.5, (f) Thickness ratio of soft rock *Hs/H* = 0.6, (g) Thickness ratio of soft rock *Hs/H* = 0.7, (h) Thickness ratio of soft rock *Hs/H* = 0.8, and (i) Thickness ratio of soft rock *Hs/H* = 0.9.

It can be seen from the above analysis that different temperatures have different degrees of influence on the mechanical properties of rock samples. Fig 12 shows the number of microcracks as a function of vertical strain for different working conditions.

As can be seen from Fig 12, there are obvious differences in the relationship between the number of microcracks and the vertical strain for different working conditions. Under the condition of soft rock thickness ratio *Hs/H* < 0.5, the relationship between the number of microcracks and the soft rock thickness ratio shows a two-stage change trend at *T* = 100°C, 200°C, and 300°C. In the first stage, the condensation between the particles is reduced due to the application of temperature, resulting in a large number of microcracks in the initial stage of compression. The second stage, the gradual development of microcracks with the increase of vertical strain. In this stage, the number of microcracks gradually increases as the vertical strain increases due to continuous compression. When the loading temperature is *T* = 400°C, the relationship between the number of microcracks and the ratio of soft rock thickness gradually changes to a two-stage trend. When the thickness ratio of soft rock is *Hs/H* = 0.1, 0.2, and 0.3, it is mainly the stage of rapid development of microcracks until the sample is destroyed. When the soft rock thickness ratio *Hs/H* = 0.4, the relationship between the number of microcracks and the soft rock thickness ratio shows a two-stage change relationship. It is mainly the initial stage of microcrack development and the stage of microcrack development with the increase of vertical strain. Under the condition that the soft rock thickness ratio *Hs/H* ≥ 0.5, the relationship between the number of microcracks and the soft rock thickness ratio presents a three-stage variation relationship under the four temperature conditions. In the first stage, the cohesion between particles is reduced due to the effect of temperature load, resulting in more microcracks in the initial compression stage. The second stage is the stage where the number of microcracks gradually increases with increasing vertical strain. In the third stage, the number of microcracks increases rapidly, and the increase in vertical strain is small. As the thickness of the soft rock increases, the trend of the third stage changes becomes more and more significant.

**Fig 13:**
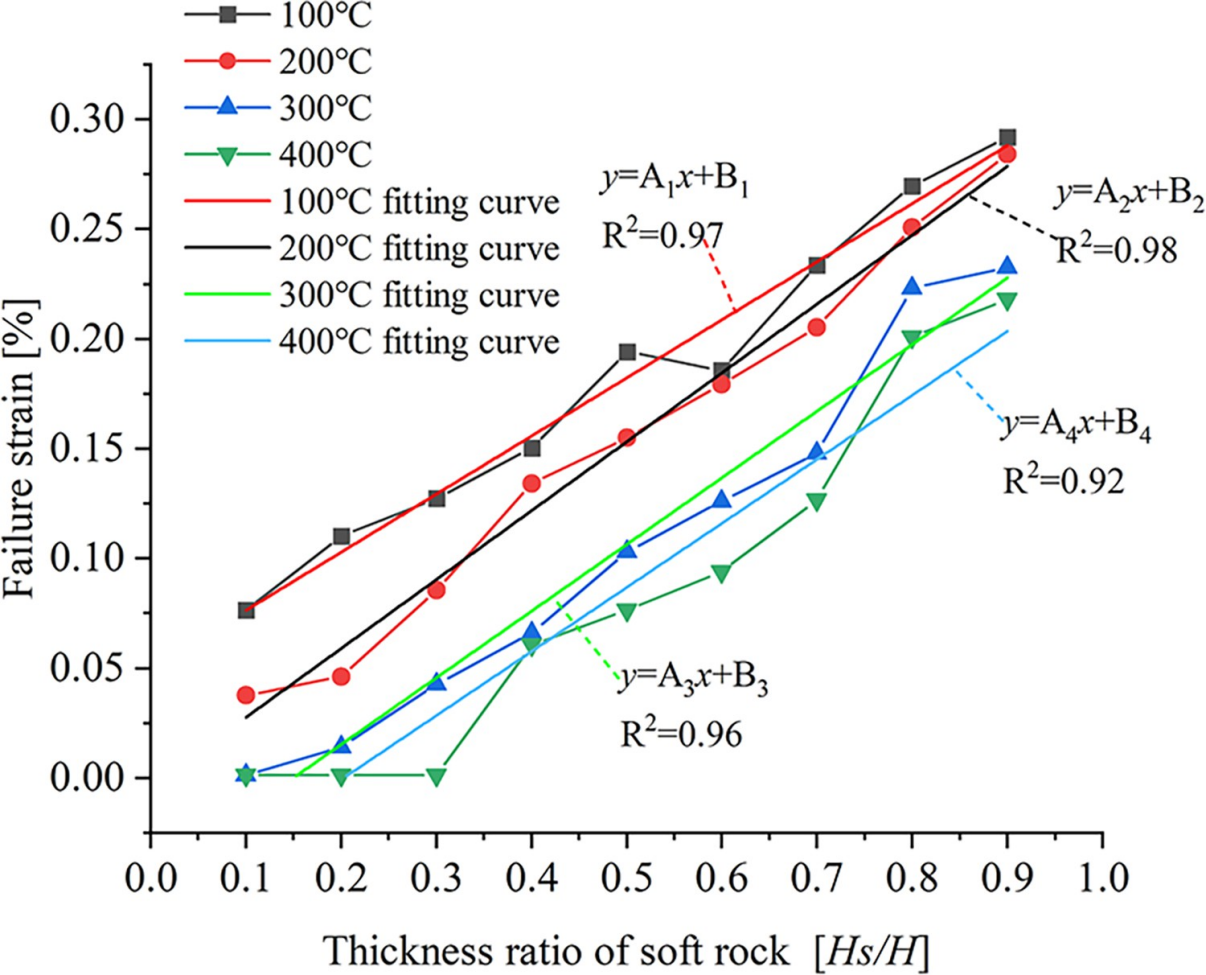
Relationship curve between failure strain and thickness ratio of soft rock.

The relationship between the failure strain and the soft rock thickness ratio under the four temperature conditions is shown in Fig 13.

It can be seen from Fig 13 that the failure strain increases gradually with the increase of the soft rock thickness ratio at the same temperature. When the loading temperature is *T* = 100°C, the failure strain has the maximum value under the condition of different soft rock thicknesses, while the failure strain is smaller when the loading temperature is *T* = 400°C. At different soft rock thicknesses, the high-temperature conditions have a more significant effect on the damage of rock samples. From the fact that the failure strain has a large value when the thickness of soft rock is large, the influence of temperature on the mechanical properties of soft rock is relatively small. The fitting curves of the relationship between failure strain and soft rock thickness ratio at different temperatures are shown in Fig 13. The fitting formula is:

$$y=A_{i}x+B_{i}$$ (7)

where, the fitting curve parameters *A*_i_ and *B*_i_ values are shown in Table 5. By obtaining the fitting curve and its parameters, the failure strain value of the rock mass sample can be approximated under other temperature conditions, and then the failure characteristics of the rock mass can be approximated.

**Table 5:** Curve fitting parameters for the relationship between failure strain and soft rock thickness ratio.

Temperature (°C)	*A*_i_(i = 1,2,3,4)	*B*_i_(i = 1,2,3,4)
100	0.26	0.050
200	0.31	-0.004
300	0.30	-0.046
400	0.29	-0.059

As can be seen from the above analysis, the temperature has a more significant effect on the mechanical properties of hard rock. Under the four temperature conditions, when the thickness of the soft rock is larger, the failure deformation of the rock mass sample is relatively less affected by the temperature.

### Analysis of failure strength

**Fig 14:**
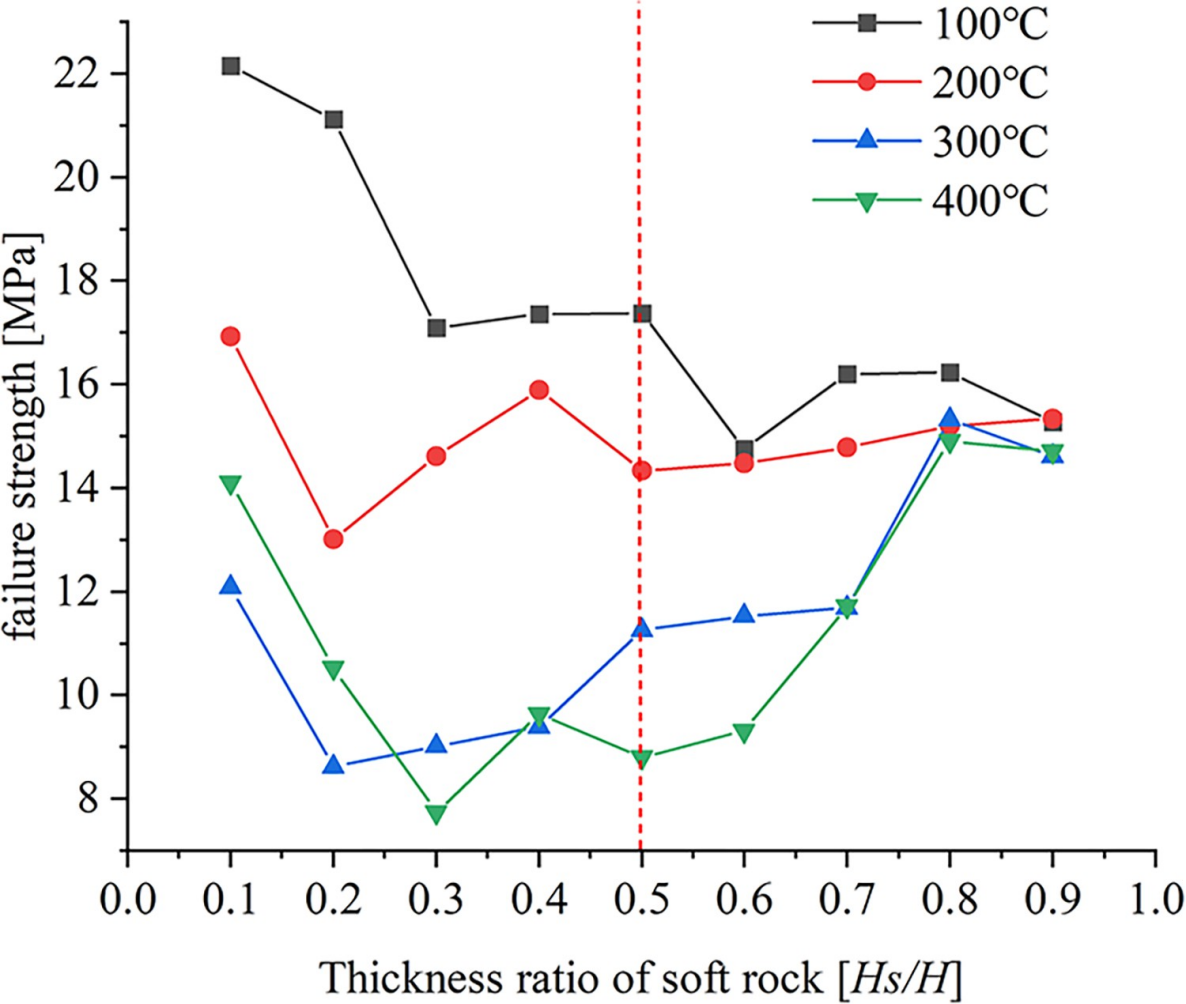
Relationship curve between failure strength and thickness ratio of soft rock.

The failure strength of the specimen obtained by uniaxial compression is quite different due to the change of the cohesive properties between the sample particles during the application of temperature. Fig 14 shows the failure strength of the specimen as a function of the soft rock thickness ratio for different working conditions.

As can be seen from Fig 14, there is a large difference in the failure strength of the samples under different working conditions. Under the condition of soft rock thickness ratio *Hs/H* < 0.5, the failure strength of the specimens generally showed a gradual decrease with the increase of soft rock thickness at *T* = 100°C and 200°C. When the loading temperature *T* = 300°C and 400°C, the failure strength of the samples first decreased and then increased with the increase of the soft rock thickness ratio. The high-temperature condition of 400°C causes the microcracks and the structural surface to coagulate, resulting in the failure strength of the samples being slightly greater than that at 300°C when the thickness ratio of soft rock is *Hs/H* = 0.1 and 0.2. Under the condition of soft rock thickness ratio *Hs/H* ≥ 0.5, the failure strength decreases gradually with the increase of soft rock thickness at *T* = 100°C, and the trend is gentle. When the loading temperature is *T* = 200°C, the failure strength increases gradually with the increase of the soft rock thickness, but the increasing trend is gentle. When the loading temperature is *T* = 300°C and 400°C, the failure strength increases gradually with the increase of the soft rock thickness. Based on the above results, it can be seen that when the thickness of soft rock is smaller than that of hard rock, high the temperature has a weakening effect on the failure strength of the sample. When the thickness of soft rock is greater than that of hard rock, the failure strength of the sample is enhanced at high temperatures.

## Discussion

In this paper, the interbedded rock mass containing microcracks with different thicknesses of soft and hard rock is analyzed. The displacement and contact force due to the applied temperature, as well as the strain, the development of microcracks, and the failure strength during the uniaxial compression stage are analyzed. As can be seen from the analysis results, high temperatures have an important effect on the mechanical properties of soft-hard interbedded rock masses with microcracks.

1. Because different rocks have different thermal expansion coefficients, the rock will produce different degrees of expansion when heated. Under the same working conditions, the displacement and contact forces due to the applied temperature gradually increase with the increase in temperature. When the soft rock thickness ratio *Hs/H* < 0.5, the large displacement area generated by the applied temperature gradually expands with the increase of the soft rock thickness ratio. At the same time, due to the influence of the structure plane on the thermal expansion of the particles, the large displacement area is mainly concentrated in the structure plane. Since the thermal expansion of hard-rock particles is strongly influenced by temperature and by the initial microcracks, large contact forces are concentrated at the ends of the initial microcracks in hard-rock regions. When the thickness ratio of soft rock *Hs/H* ≥ 0.5, the large displacement area gradually decreases with the increase of the thickness ratio of soft rock due to the small thermal expansion of soft rock particles. The zone of large displacement affected by the initial microcrack is concentrated on either side of the initial microcrack. The structural plane has little effect on the large displacement distribution. Due to the smaller thermal expansion properties of soft rock particles at high temperatures, the contact forces generated in soft rock regions are smaller. The contact force in hard rock areas decreases with the increase of soft rock thickness. Both the peak displacement and the peak contact force generated under the four temperature conditions first increase and then decrease with increasing soft rock thickness, with the trend becoming more significant for higher temperatures. When the thickness of soft rock is large, the peak displacement is less affected by the position of the structural plane. When an initial microcrack penetrates the structural plane of soft and hard rock, the contact force is strongly influenced by temperature.
2. The cohesion between hard rock particles is reduced due to high temperature, which leads to more significant development of microcracks in the hard rock area. When the initial microcracks are located in regions of hard rock, the development of microcracks is less affected by the initial microcracks and the hard rock is more broken. Because the bonding properties between soft rock particles are less affected by temperature, the microcracks begin to expand from the end of the initial microcracks. When the thickness ratio of soft rock *Hs/H* < 0.5, the influence on the bonding force of soft rock and hard rock are relatively small at *T* = 100°C, and it is affected by the initial microcracks and the structural surface. As a result, the number of microcracks first decreases and then increases with the increase of the soft rock thickness ratio. The bonding properties between soft and hard rock particles are greatly affected at *T* = 200°C, 300°C, and 400°C, so the number of microcracks gradually decreases with the increase of soft rock thickness. Because high temperature has little effect on the cohesion between soft rock particles, the number of microcracks generated in the compression stage gradually decreases with the increase of soft rock thickness when the soft rock thickness ratio *Hs/H* > 0.5.
3. The bonding properties between hard rock particles are greatly affected by temperature. When the thickness ratio of soft rock *Hs/H* < 0.5, the relationship between the number of microcracks and the vertical strain showed two-stage changes at *T* = 100°C, 200°C, and 300°C. In the first stage, the microcracks develop rapidly during the initial stage of compression. In the second stage, the number of microcracks increases as vertical strain increases due to continuous compression. When the loading temperature *T* = 400°C, the relationship curve gradually develops from the rapid increase of the number of microcracks in the initial stage to the gradual development stage of microcracks with the increase of vertical strain. Because the bonding properties between soft rock particles are less affected by temperature, the thickness of soft rock gradually increases. As a result, when the soft rock thickness ratio is *Hs/H* ≥ 0.5, the relationship between the number of microcracks and the vertical strain changes in three stages. In the first stage, the microcracks develop rapidly during the initial compression stage due to the effect of the early temperature load. The second stage is the phase in which microcracks gradually develop with increasing vertical strain. In the third stage, the number of microcracks increases rapidly in the later stage of compression, while the vertical strain changes little. However, the changing trend of the third stage is gradually significant for the four temperature conditions. The larger the thickness of the soft rock, the greater the failure strain under the four temperature conditions, and the high temperature aggravates the damage of the sample rock mass.
4. Due to the high temperature, the cohesive force between the hard rock particles is reduced, resulting in a large difference in the failure strength of the sample. When the thickness ratio of soft rock is *Hs/H* < 0.5, the failure strength of the specimen decreases gradually with the increase of the thickness ratio of soft rock at *T* = 100°C and 200°C. Because the high temperature strengthens the cohesive force between the soft rock particles, the failure strength of the samples generally shows a trend of first decreasing and then increasing with the increase of the thickness of the soft rock at *T* = 300°C and 400°C. When the soft rock thickness ratio is *Hs/H* ≥ 0.5, because the high temperature has a strengthening effect on the bonding property between soft rock particles, the soft rock thickness gradually increases. As a result, the failure strength of the sample gradually decreases when the loading temperature is *T* = 100°C. However, the failure strength of the samples increases gradually with the increase of the soft rock thickness when the loading temperature is *T* = 200°C, 300°C, and 400°C. Overall, it can be seen that the high-temperature conditions have a weakening effect on the strength of failure of the sample when the thickness of the soft rock is small. When the thickness of soft rock is larger, the high-temperature conditions have a strengthening effect on the failure strength of the sample.
5. In this paper, the failure characteristics of the interbedded rock mass with microcracks are analyzed under working conditions. For real rock masses, the strength of rock mass is affected by many factors. In the face of deep resource extraction and related engineering construction in different formation conditions, it is important to investigate in depth the influence of different influence factors or multi-factor superpositions on the strength properties of rock masses.

## Conclusion

In this paper, the particle flow program (PFC^2D^) is used to establish a numerical test of uniaxial compression considering the effect of temperature loading. And the rationality of the temperature loading method is verified. On this basis, the mechanical properties of the soft-hard interbedded rock masses with microcracks were analyzed under different temperature conditions. The displacement and contact forces generated during the temperature application stage, as well as the microcrack development, vertical strain, and damage strength during the uniaxial compression stage are analyzed in depth. The main conclusions reached are as follows:

1. Under the same thickness of soft rock, the displacement influence zone generated by applying temperature gradually expands with the increase of temperature. When the soft rock thickness ratio *Hs/H* < 0.5, the large displacement area caused by the applied temperature is concentrated at the structural plane. However, the large contact force is mainly concentrated at the end of the initial microcrack. When the thickness ratio of soft rock is *Hs/H* ≥ 0.5, the large displacement area caused by the applied temperature is concentrated on both sides of the initial microcracks and the displacement influence area decreases with the increase of the thickness of soft rock. In contrast, the contact force is concentrated in the hard rock area. The relationship between the peak displacement and the thickness ratio of soft rock is a quadratic function, first increasing and then decreasing. The larger the thickness of the soft rock, the smaller the value of the peak displacement. When the soft rock thickness ratio *Hs/H* < 0.4, the peak contact force generated by the applied temperature gradually increases with the increase of the soft rock thickness ratio, and the higher the temperature, the more significant the increase trend. When the soft rock thickness ratio *Hs/H* ≥ 0.4, the peak contact force gradually decreases with the increase of the soft rock thickness ratio.
2. When the soft rock thickness ratio *Hs/H* < 0.5, the microcracks produced by the sample failure are more significant in the hard rock area. The initial microcracks have little effect on the crack development during the failure stage of the specimen. When the applied temperature *T* = 100°C, the number of microcracks produced by the failure of the specimen decreases first and then increases with the increase of the thickness ratio of soft rock. When the applied temperature *T* = 200°C, 300°C and 400°C, the number of microcracks gradually decreases with the increase of the thickness ratio of soft rock. When the soft rock thickness ratio *Hs/H* ≥ 0.5, the microcracks in the soft rock area gradually develop from the end of the initial microcracks. The number of microcracks generated in the four temperature conditions gradually decreases with increasing soft rock thickness ratio. When the thickness of hard rock is large, it is greatly affected by temperature, and the sample is broken after uniaxial compression.
3. The relationship between the number of microcracks and vertical strain under different temperature conditions is quite different. When the thickness ratio of soft rock is *Hs/H* < 0.5, the relationship between the number of microcracks and the vertical strain is mainly changed in two stages when the temperature is *T* = 100°C, 200°C and 300°C. In the first stage, microcracks develop rapidly in the initial stage of compression due to temperature loading. In the second stage, the microcracks gradually increase with increasing vertical strain. When the applied temperature *T* = 400°C and the soft rock thickness ratio *Hs/H* ≤ 0.2, the micro-cracks increase rapidly and the vertical strain is small until the sample is destroyed. When the thickness ratio of soft rock is 0.2 ~ 0.5, the relation curve shows a two-stage change trend. When the thickness ratio of soft rock is *Hs/H* ≥ 0.5, the relationship between the number of microcracks and the vertical strain changes in three stages. The initial compression stage microcrack rapid generation stage, microcrack increases gradually with the increase of vertical strain, and the rapid development stage of micro-cracks in specimen failure stage. High temperatures aggravate the deformation and failure of rock masses at different thickness of soft rock.
4. When the thickness ratio of soft rock is *Hs/H* < 0.5, the failure strength of the specimen decreases with the increase of the thickness of soft rock when the applied temperature *T* = 100°C and 200°C. However, when the applied temperature *T* = 300°C and 400°C, the failure strength of the specimen decreases first and then increases with the increase of the thickness of soft rock. Under the condition that the thickness ratio of soft rock *Hs/H* ≥ 0.5, when the applied temperature *T* = 100°C, the failure strength gradually decreases with the increase of the thickness of soft rock, and the decreasing trend is relatively gentle. When the applied temperature *T* = 200°C, 300°C and 400°C, the failure strength gradually increases with the increase of the thickness of soft rock. The increasing trend is relatively gentle when the applied temperature *T* = 100°C. When the thickness of the soft rock is large, the high temperature conditions can strengthen the failure strength of the specimen.
